# Biological treatment of ankylosing spondylitis: a nationwide study of treatment trajectories on a patient level in clinical practice

**DOI:** 10.1186/s13075-019-1908-9

**Published:** 2019-05-28

**Authors:** Ulf Lindström, Tor Olofsson, Sara Wedrén, Ilia Qirjazo, Johan Askling

**Affiliations:** 1 Clinical Epidemiology Division, Department of Medicine, Karolinska Institutet, Stockholm, Sweden; 2 Department of Clinical Sciences Lund, Lund University, Lund, Sweden; 3 Rheumatology Department, Linköping University Hospital, Linköping, Sweden; 4 Clinical Epidemiology Unit, Karolinska University Hospital Solna, Stockholm, Sweden

**Keywords:** Ankylosing spondylitis, Tumour necrosis factor inhibitor, Treatment, Observational

## Abstract

**Background:**

There is substantial evidence that patients with ankylosing spondylitis (AS) have high response rates to tumour necrosis factor inhibitors (TNFi), a low likelihood of successful treatment termination, but yet a limited drug retention. Whereas several reports have assessed drug retention rates for TNFi in AS, there are few, if any, studies investigating the actual treatment trajectories on a patient level, including subsequent therapy changes and dose reductions, of individual patients. The aim of this study was to describe 5-year treatment trajectories in patients with ankylosing spondylitis (AS) starting a first TNFi.

**Methods:**

Bio-naïve patients with AS starting a TNFi in 2006–2015 were identified in the nationwide Swedish Rheumatology Quality register and followed until 31 December 2015. All changes in their anti-rheumatic treatment during follow-up were recorded. To further increase precision, these data were complimented by information on the amount of prescribed subcutaneous TNFi collected from pharmacies during each year, retrieved from the Swedish Prescribed Drug Register.

**Results:**

Two thousand five hundred ninety patients started a first TNFi 2006–2015, and after 1 year, 74% remained on their first TNFi. However, after 5 years, this figure was only 46%, although at that time 63% were still on treatment with any biologic, while 30% had no anti-rheumatic treatment at all. After discontinuing the first TNFi, 46% switched directly to a second TNFi, but the drug retention for the second and third TNFi grew successively shorter compared to that for the first TNFi. In contrast, patients remaining on treatment with their first subcutaneous TNFi gradually reduced the dose, so that during the fifth year of treatment only 66% had collected ≥ 75% of the defined daily doses for that year.

**Conclusion:**

Less than half of patients with AS will remain on their first TNFi after 5 years, but most are still on a biologic. While patients remaining on treatment with their first TNFi appear to be able to reduce the dose over time, a large proportion cycle through several biologics, and 1/3 have no anti-rheumatic treatment after 5 years. This indicates the importance of thorough follow-up programs as well as a need for alternative therapeutic options.

**Electronic supplementary material:**

The online version of this article (10.1186/s13075-019-1908-9) contains supplementary material, which is available to authorized users.

## Background

Following approval of the first tumour necrosis factor alpha inhibitor (TNFi) for use in ankylosing spondylitis (AS) 15 years ago, TNFi treatment has become the mainstay treatment for active AS [1, 2]. The main reason for this is the high response rate, where randomized controlled trials (RCTs) have suggested that up to 30% of patients with axial spondyloarthritis (SpA) or AS may even achieve inactive disease, defined as ASDAS (ankylosing disease activity score) below 1.3, at 24 weeks after treatment start [3, 4].

A substantial number of observational studies have described TNFi drug retention in AS from various perspectives, searching for predictors for remaining on treatment, and comparing retention rates in different subgroups [5–25]. These long-term observational data describe a somewhat different picture than that of the RCTs, suggesting that 1 year after starting a first TNFi around 80% remain on this treatment. After 2 years, 60–70% remain on treatment and after 5 years only every other patient still remain on their initial TNFi. Observational studies have also shown that AS patients failing a first TNFi may benefit from a second [14, 26–29], but that the drug retention of a second TNFi is inferior to that of the first TNFi, in particular among individuals switching due to primary lack of efficacy [11, 30]. At the same time, studies have indicated that while TNFi dose reduction may be feasible in patients who have reached low disease activity, most patients who discontinue a TNFi altogether will relapse [31, 32]. Table 1 summarizes seminal studies that have assessed TNFi retention rates and dose reduction in AS.

**Table 1:** Seminal studies describing TNFi drug retention rates and TNFi dose reduction, tapering or discontinuation

Author	No. AS patients	TNFi drug retention rate
Carmona et al. 2006 [5]	657	1 year 88%, 2 years 82%, 3 years 76%
Heiberg et al. 2008 [8]	249	1 year 78%
Pavelka et al. 2009 [7]	310	1 year 84%, 2 years 76%, 3 years 72%
Glintborg et al. 2010 [9]	842	1 year 74%, 2 years 63%
Kristensen et al. 2010 [16]	243	2 years 74%
Lie et al. 2011 [14]	514	1 year 76%, 2 years 65%
Arends et al. 2011 [12]	220	1 year 71%, 2 years 66%
Arends et al. 2012 [13]	111	3 years 65%
Glintborg et al. 2013 [11]	1436	2 years 58%
Heinonen et al. 2015 [15]	543	1 year 84%, 2 years 75%
Lorenzin et al. 2015 [17]	70	1 year 77%, 2 years 70%, 3 years 57%, 4 years 53%, 5 years 50%
Arends et al. 2017 [33]	89	7 years 51%
Author	No. AS patients	TNFi discontinuation or reduction
Baraliakos et al. 2005 [31]	42	91% relapse 36 weeks after infliximab discontinuation
Brandt et al. 2005 [32]	26	> 2/3 relapse 12 weeks after etanercept discontinuation
Zhao et al. 2018 [34]	35	60% relapse 3 years after etanercept discontinuation
Lee et al. 2010 [35]	109	Etanercept dose reduction may be possible
De Stefano et al. 2014 [36]	38	Etanercept reduction may be possible at clinical remission
Cantini et al. 2013 [37]	78	Etanercept reduction may be possible at clinical remission
Yates et al. 2015 [38]	89	Etanercept dose reduction may be possible
Zavada et al. 2016 [39]	136	TNFi reduction may be possible at low disease activity
Park et al. 2016 [40]	165	TNFi reduction linked to more rapid radiographic progression
Fong et al. 2016 [41]	125	TNFi reduction may be possible at low disease activity

*AS* ankylosing spondylitis, *TNFi* tumour necrosis factor alpha inhibitor

Taken together, reports of this type underscore the need for a better understanding of the somewhat contradictory evidence that patients with AS have high response rates to TNFi, a low likelihood of successful termination of the treatment, and yet a limited drug retention. Whereas several studies have assessed drug retention rates for TNFi in AS, in particular for the first and second line TNFi, few if any studies have investigated the actual treatment trajectories on a patient level, i.e. the choice(s) of treatment and stay-time(s) for any subsequent treatments.

We set out to describe the treatment trajectories in patients with AS starting a first TNFi in clinical practice during a period of 10 years. Secondary objectives were to compare drug retention for the first, second and third TNFi and to assess the evidence for dose reduction of TNFi over time, in patients remaining on a stable treatment.

## Methods

### Study design

This is a national register-based study on bio-naïve Swedish patients with AS starting a first TNFi during a 10-year period, 2006 through 2015.

### Data sources

Data on subjects, disease activity measures, TNFi initiation and discontinuation, as well as reason for discontinuation, were collected from the Swedish Rheumatology Quality Register (SRQ). The SRQ has an estimated national coverage of 86% for patients with SpA treated with biological disease-modifying anti-rheumatic drugs (bDMARD) in Sweden [42].

Data on prescribed drugs were collected from the national Prescribed Drugs Register, which contains information such as anatomic therapeutic chemical (ATC) codes [43] and doses on all prescriptions collected at a pharmacy in Sweden since July 2005. Demographic data, such as death or migration, were retrieved from the national Population Register, and data on comorbidities, for characterizing the patient cohort, from the national Patient Register. The national Patient Register collects data such as diagnoses and procedures from inpatient care, and visits in outpatient specialized care. The coverage for inpatient care in Sweden is close to 100% and around 80% for visits in outpatient specialized care [44].

### Case definition

We identified all patients with a registered diagnosis of AS, starting a first ever TNFi between 1 January 2006 through 31 December 2015, in the SRQ. From this cohort, a subset was identified for a sensitivity analysis, including all patients starting their first TNFi between 1 January 2006 and 31 December 2010, where all patients thus had a minimum of 5 years of possible follow-up time until the end of the study period, 31 December 2015.

Data on concomitant treatment with conventional synthetic DMARDs (csDMARDs), for characterization at baseline, were retrieved from the SRQ. Use of non-steroidal anti-inflammatory drugs (NSAIDs) was defined as having collected a prescription of NSAIDs in a pharmacy during the year prior to TNFi start and was collected from the Prescribed Drug Register. Having a history of anterior uveitis or inflammatory bowel disease was defined as having been registered with these diagnoses in specialized care in the national Patient Register, at any time before starting the first TNFi. Since psoriasis is often managed in primary care (generally not covered by the national Patient Register), a history of psoriasis was defined as having either received such a diagnosis in specialized care, or complementary by having collected a prescription of anti-psoriatic drugs (ATC codes: D05) at any time-point before starting the first TNFi.

### Follow-up, censoring and dose tapering

For the primary objective of describing treatment trajectories, the treatment status after each full 12-month period since therapy initiation was recorded for each patient. Hence, at every 12 months since starting the first TNFi, we determined if the patient remained on this treatment, had switched to another bDMARD treatment, had discontinued DMARD treatment altogether or had been censored. Censoring occurred at whichever came first of death, migration, 31 December 2015 or “uncertain treatment status”. Uncertain treatment status was defined as either not having a registered visit in the SRQ in 2 years, or, for patients on a subcutaneous TNFi, not having collected a prescription of their medication from a pharmacy in 6 months.

For the secondary objective of comparing drug retention for the first, second and third TNFi, the follow-up time was defined by the start date and stop date of the TNFi, and censoring was performed as described above, with the addition of censoring at discontinuation of the TNFi due to pregnancy or remission/inactive disease.

For the other secondary objective of assessing if dose reduction occurred over time, the number of defined daily doses (DDDs) collected at a pharmacy during each 12-month period since treatment initiation was retrieved from the Prescribed Drug Register for each of the subcutaneous TNFi. Infliximab was not included in this assessment since comparable data on dose reduction/tapering were not available through the same data source. For all patients remaining on their first TNFi, at each 12-month interval of treatment, the proportion collecting at least 75% of the yearly DDDs in the last year of treatment was calculated. To avoid including patients who had either been lost to follow-up (dead or migrated), or with an uncertain treatment status, the same censoring principles were applied as described above.

### Statistics

To compare drug retention rates for the first, second and third TNFi, hazard ratios (HR) with 95% confidence intervals (95%CI) for drug discontinuation were assessed through Cox proportional hazard analyses, also adjusted for sex and age at start of the respective TNFi. A marginal Cox model was used to calculate robust confidence intervals for the HR, in order to accommodate for clustering (the same patients contributing to more than one treatment episode). The assumption of proportional hazards was tested though inserting an interaction term between follow-up time and each exposure at a time, and through visual assessment of the survival curves and log-minus-log survival plots. Statistical significance of baseline trends (e.g. number of patients starting TNFi yearly 2006–2015), and dose tapering, was determined through linear regression analyses. All statistical analyses were performed using SAS version 9.4.

Treatment trajectories, focusing on alterations in bDMARD treatment and employing the same censoring principles as described above, were illustrated using Sankey diagrams. Two Sankey diagrams were drawn, the first depicting the drug-specific trajectories for all AS patients starting and discontinuing their first TNFi during the study period (2006–2015) (i.e. changing their bDMARD treatment trajectory the first time, e.g. switching from infliximab to adalimumab). The second Sankey diagram depicted the subgroup of patients changing their bDMARD treatment trajectory also a second time during the study period (e.g. first switching from infliximab to adalimumab and then discontinuing adalimumab due to pregnancy). Each diagram displays four potential states after discontinuation: (1) switching to another bDMARD within 180 days of discontinuing the first, (2) remaining without bDMARD treatment or having a gap in bDMARD treatment for more than 180 days (or up until censoring), (3) discontinuing and remaining without bDMARD due to pregnancy (regardless of remaining without treatment for more or less than 180 days), and (4) discontinuing and remaining without bDMARD due to disease remission (regardless of remaining without treatment for more or less than 180 days). The Sankey diagrams thus only include patients with the specific requirement of starting and changing treatment once or twice within 2006–2015 and exclude any changes made after 31 December 2015, or after censoring.

### Ethical approval

The ethical review board in Stockholm, Sweden, approved the study (dnr:2011/29-31/1).

## Results

Between 1 January 2006 and 31 December 2015, 2590 AS patients started a first ever TNFi, for demographics and baseline characteristics see Table 2. At baseline, 24% had concomitant csDMARD treatment, 82% were on treatment with NSAIDs and 18% had a mixed phenotype including peripheral disease (defined as ≥ 1 swollen joint recorded during the year prior to TNFi start). There was a gradual increase in number of new-starts per year, from 161 in 2006 to 278 in 2015 (*p* value 0.0036), but there were no statistically significant calendar trends in age, disease duration, BASDAI (Bath Ankylosing Spondylitis Disease Activity Index) or ASDAS at start of the first TNFi, although there was a significant trend for decreasing CRP over time, as described in detail in Additional file 1. The CRP trend may suggest that the increase in new-starts is at least partly explained by a lower threshold for initiating bDMARDs in clinical practice. As a reference in Additional file 1 is also provided the number of patients with rheumatoid arthritis starting a first ever bDMARD during the same time-period (2006–2015), indicating a similar but less pronounced calendar-year increase [45].

**Table 2:** Baseline characteristics of bio-naïve AS patients starting a first TNFi in 2006–2015

Baseline characteristics	2006–2015 cohort	2006–2010 subset-cohort
Total number	2590	1167
Sex, men (%)	1827 (71)	838 (72)
Age, mean (sd)	44 (13.2)	44 (12.8)
Disease duration	16 (12.3)	16 (11.8)
csDMARD (concomitant use), *n* (%)	634 (24)	384 (33)
NSAID (concomitant use), *n* (%)	2136 (82)	972 (83)
Peripheral disease^a^	464 (18)	195 (17)
Type of TNFi		
Infliximab, *n* (%)	910 (35)	478 (41)
Adalimumab, *n* (%)	782 (30)	405 (35)
Golimumab, *n* (%)	329 (13)	9 (1)
Etanercept, *n* (%)	483 (19)	273 (23)
Certolizumab pegol, *n* (%)	86 (3)	2 (0)
Extra-articular SpA manifestations		
Anterior uveitis, *n* (%)	711 (27)	308 (26)
Psoriasis, *n* (%)	156 (6)	65 (6)
Inflammatory bowel disease, *n* (%)	179 (7)	95 (8)

^a^≥ 1 swollen joint recorded at a visit in the year prior to TNFi start

*csDMARDs* conventional synthetic disease-modifying anti-rheumatic drugs, *NSAID* non-steroidal anti-inflammatory drugs, *TNFi* tumour necrosis factor alpha inhibitors, *SpA* spondyloarthritis

### Treatment trajectories

**Fig. 1:**
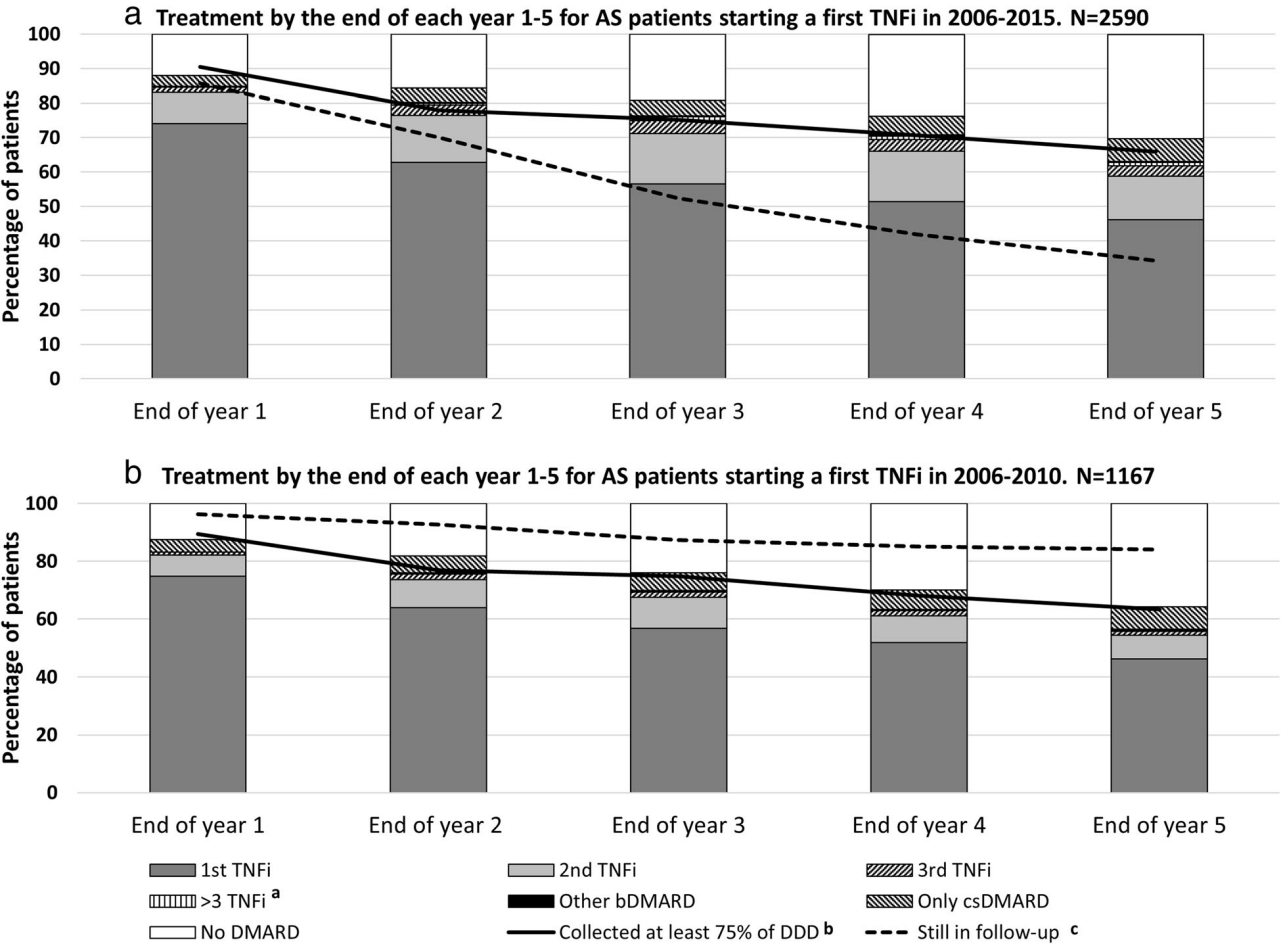
**a**, **b** Current treatment after one to five whole years since starting a first TNFi. The figure describes the treatment status for bio-naïve patients with ankylosing spondylitis, 1–5 years after starting a first ever TNFi. (a) Patients who have either used > 3 different TNFi or cycled back to previous TNFi. (b) The solid black line indicates the percentage of the patients who are still on their first TNFi at the end of each year, and who have collected at least 75% of that year’s defined dose from a pharmacy. (c) The dotted black line indicates the percentage who were lost to follow-up (censored) due to death, emigration, “uncertain treatment status (see Methods)” or past the end of the study period. DDD = defined daily dose; csDMARDs = conventional synthetic disease-modifying anti-rheumatic drugs; TNFi = tumour necrosis factor alpha inhibitor; AS = ankylosing spondylitis

Figure 1 presents the treatment status at the end of each full 12-month period counting from the start date of the first TNFi, among patients initiating TNFi in 2006–2015 (Fig. 1a), and in the subset with a potential for at least 5 years of follow-up, starting in 2006–2010 (Fig. 1b).

In the whole cohort of patients (starting in 2006–2015), 46% were still on their first TNFi, 13% on their second TNFi, 7% only on csDMARDs and 30% had no DMARD treatment at the end of 5 years. Furthermore, 45% of the cohort met the end date of the study (31 December 2015) before being followed 5 years, and a further 20% were censored for other reasons (18.5% due to uncertain treatment status [only contributing in total to 11% decrease of the total person-time] and 1.5% migrated/died).

In the subset starting treatment 2006–2010, only 16% had been censored before reaching five whole years of follow-up time (13% due to uncertain treatment status and 3% due to migration or death). Similar to the whole cohort, 46% of patients in this subset remained on their first TNFi after 5 years, 8% on their second TNFi, 8% only on csDMARD and 36% had no DMARD treatment.

**Fig. 2:**
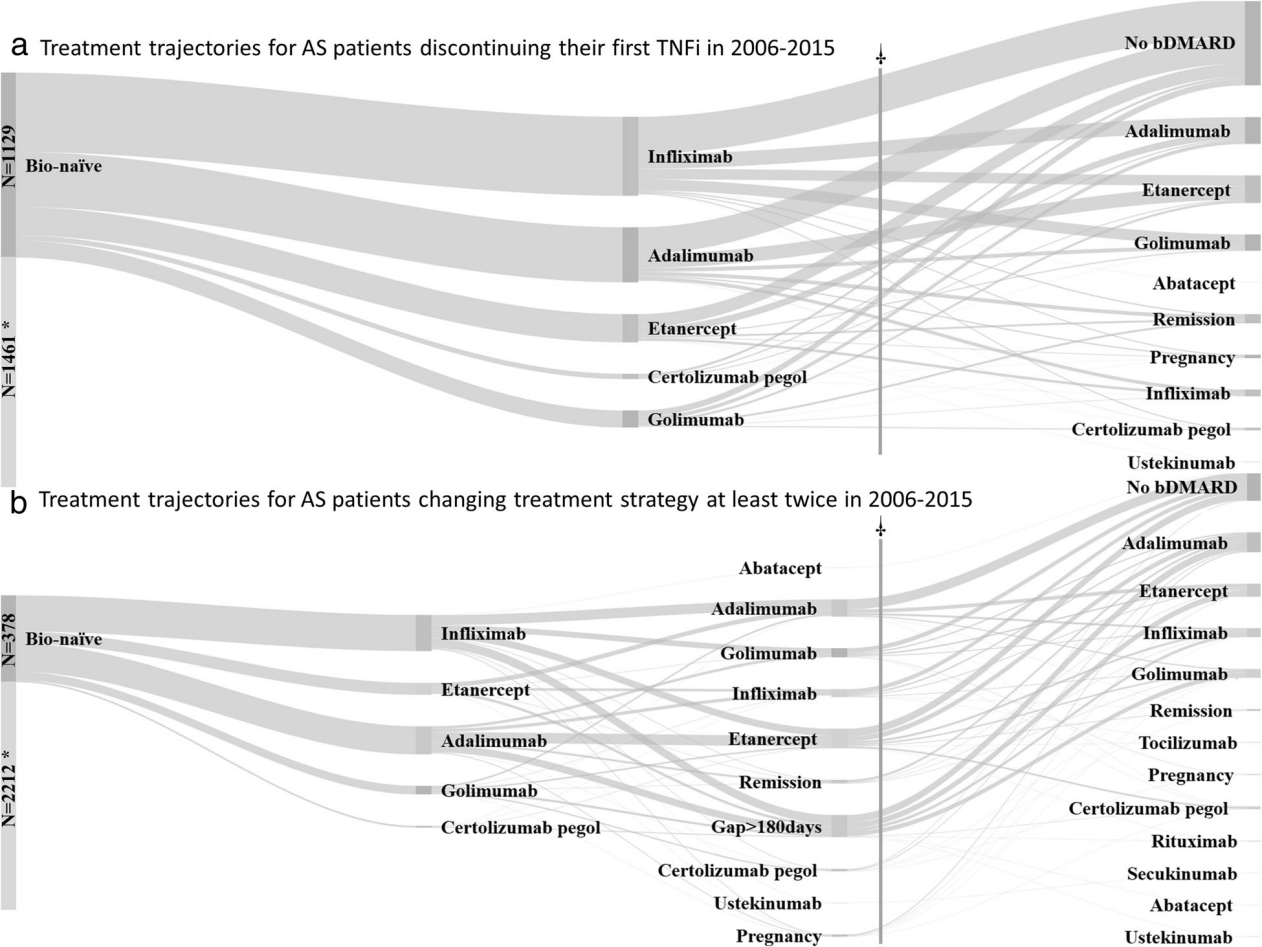
Treatment trajectories for bio-naïve AS patients starting a first TNFi in 2006–2015. **a** The trajectories for all patients discontinuing their first TNFi within the study period 2006–2015 and prior to censoring (*N* = 1129). **b** All patients changing their treatment trajectory also a second time within the study period (*N* = 378). TNFi = tumour necrosis factor alpha inhibitor; AS = ankylosing spondylitis

Figure 2 illustrates the drug-specific treatment trajectories for patients switching treatment strategy once (Fig. 2a, *N* = 1129) or twice (Fig. 2b, *N* = 378) during 2006–2015. As can be seen in Fig. 2a, a substantial proportion of the patients who discontinue their first TNFi do not immediately switch to another treatment (47%). The reasons for discontinuation were listed as follows: adverse effects 27%, primary ineffectiveness 20%, secondary ineffectiveness 19%, “other” 26%, missing 1%. Further, only a small minority of patient who discontinue bDMARD treatment a first or second time do so because of attaining disease remission (5% and 2%, respectively). However, as can be seen in Fig. 2b, around 30% of those who change treatment strategy a second time are patients who restart bDMARD treatment after a gap of more than 180 days, or after bDMARD-free disease remission. This in turn suggests that a large proportion of the patients who discontinue their first TNFi for a longer period, regardless of reason, later had to start treatment again. Patients shifting treatment strategy a second time also utilized a wider range of bDMARDs as their subsequent treatment.

### Treatment persistence

**Fig. 3:**
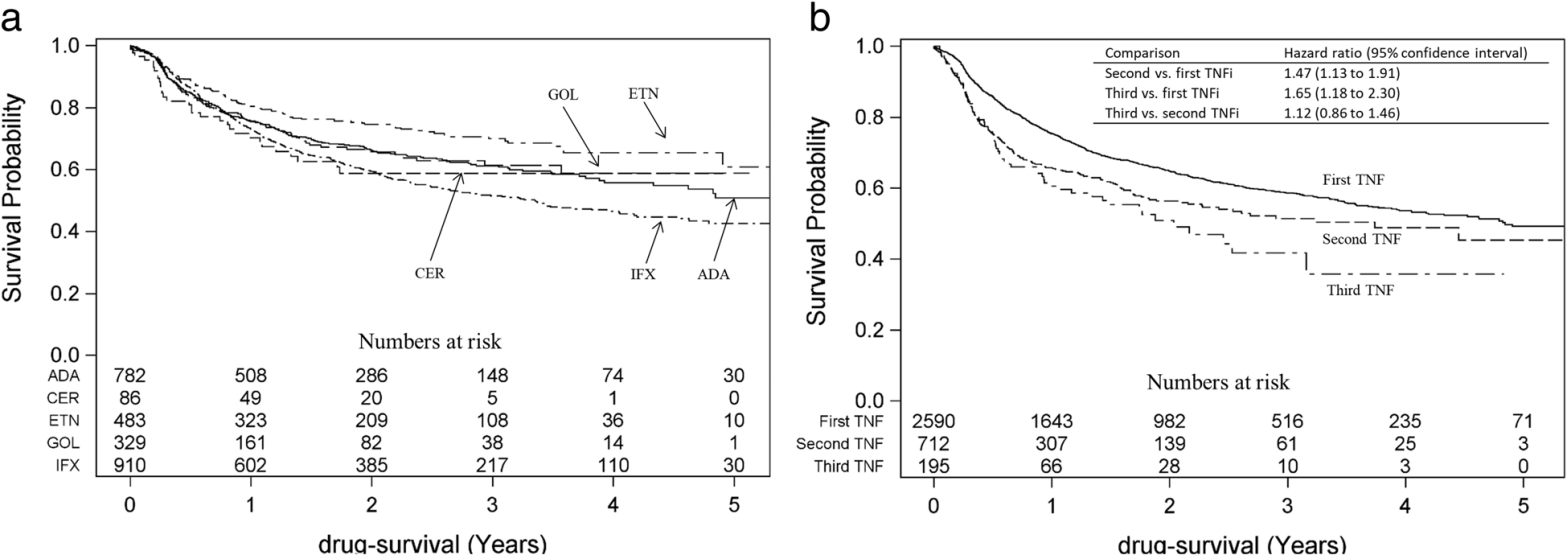
Survival probability plots, for drug retention in patients with ankylosing spondylitis starting a first TNFi 2006–2015. **a** Comparing the five different available TNFi, used as the first ever TNFi. **b** Comparing the first, second and third TNFi, with hazard ratios for TNFi discontinuation also adjusted for sex and age at the respective treatment start. TNFi = tumour necrosis factor alpha inhibitor; ADA = adalimumab; CER = certolizumab pegol; ETN = etanercept; GOL = golimumab; IFX = infliximab

Figure 3a illustrates the survival curves for the five available TNF inhibitors when used as the first bDMARD (2006–2015). The drug retention for the subcutaneous TNF is were similar, while patients discontinued infliximab at a higher rate.

Figure 3b presents the survival curves for the first, second and third TNFi (2006–2015). The 5-year drug retention was higher for the first TNFi, compared to the second TNFi, and markedly lower for the third TNFi. The survival curves further indicated a more pronounced discontinuation rate for the second TNFi during the first year after switch of TNFi, compared to the first TNFi, but with a diminishing difference later on. Figure 3b also presents the age- and sex-adjusted HR for discontinuing the first, second and third TNFi, with a HR of 1.47 (95% CI 1.13 to 1.91) for discontinuing the second TNFi as compared to the first TNFi, and 1.65 (95%CI 1.18 to 2.30) for the third TNFi as compared to the first. The assumption of proportional hazards was however rejected, for the comparison between the first and second TNFi, based on the converging appearance of the survival curves and on a statistically significant interaction term between the follow-up time and the exposure. Through testing different cut-off points for the follow-up time, it was determined that the optimal cut-off was around 1 year. Stratifying the follow-up time on < 1 year and ≥ 1 year, indicated that all of the increased risk of discontinuing the second TNFi occurs in the first year: HR 1.51 (95%CI 1.30 to 1.76) in the first year, compared to HR 0.91 (95% CI 0.67 to 1.24) after the first year.

### Dose reduction

In Fig. 1a and b, the solid black line indicates the percentage of patients, still on their first subcutaneous TNFi at the end of each year, who at the end of each full year of treatment have collected ≥ 75% of the DDDs for a full year of treatment. Overall, for the first 5 years of treatment, there was a gradual drop from 90% after the first year, to 66% after the fifth year, with a *p* value for the trend of 0.008, while for the 2006–2010 subset, the corresponding numbers were 89 to 63% (*p* value 0.005).

## Discussion

Our nationwide, population-based, study indicates that half of the bio-naïve patients with AS who start a first TNFi will discontinue this treatment within the following 5 years. In fact, only 46% remained on their first TNFi after 5 years, another 13% on their second TNFi and 3% on their third TNFi, although the majority (63%) were still on a biologic. We also demonstrate that around half of the patients who discontinue their first TNFi switch directly to a second TNFi and that the 5-year drug retention is higher for the first TNFi, as compared to the second and the third TNFi. Interestingly, practically all of the 50% risk increase of discontinuing the second TNFi, as compared to the first, occurs in the first year of treatment. Furthermore, we found that dose reduction of the first TNFi is common in patients remaining stable on treatment over time.

To our knowledge, this is the first study specifically focusing on bDMARD treatment trajectories in AS, with a patient- rather than group-level comparison of drug retention rates. Consequently, in this regard, there is a lack of comparable studies. In support of the validity of our results, the drug retention of the first TNFi in AS are in line with previous studies, where the 2-year survival, for a first TNFi, has been reported to be 60–70% (63% in the current study) [9, 11, 14] and the 5-year survival around 50% [17]. Our results demonstrating successively lower survival rates for the second and third TNFi are also similar to previous studies [11, 14], which is also true for the shorter drug retention observed for infliximab, compared to the other TNFi. [15]. A substantial proportion of patients (24%) in our study were also treated with a csDMARD, which is likely explained by a tradition in the Nordic countries to use concomitant csDMARDs in AS, even in patients without a peripheral arthritis phenotype [46].

The population-based approach, using registers with a high rate of coverage, should give our results a good generalizability, particularly in relation to healthcare systems with a similar high patient accessibility to bDMARD treatment as in Sweden. However, a number of limitations should also be acknowledged. First, there is a considerable inter-country variability in TNFi drug retention, which can be deduced from Table 1, and probably also a calendar-time variability, warranting caution when extrapolating the results. Second, register-based studies are subjected to a limitation in terms of possible misclassification. For example, the patients are identified based on a clinical diagnosis of AS recorded in the SRQ by the treating physician. Further validation of the AS diagnosis could not be performed within the SRQ setting due to limited availability of radiographic data in the register, and it can hence not be ruled out that the current cohort may also contain some patients with non-radiographic axial SpA. However, in this study, it is unlikely that this would introduce any systematic error that would bias the results. Third, in the comparisons of drug retention rates between first, second and third TNFi, there are several potential confounders that can effect both the change in therapy itself and the outcome, which were not available for adjustment in the present setting. A number of studies have attempted to identify predictors for switching TNFi in AS [11, 14, 30, 47], but as this was not the objective of this study, this has not been investigated further. Moreover, correlating the individual patient treatment trajectories with the corresponding treatment effectiveness would be interesting, but also this was beyond the scope of the current study. Fourth, per definition, a high proportion (66%) of patients in the cohort were censored before they had reached 5 years of follow-up, decreasing the precision of the results towards the end of the study. However, in the 2006–2010 subset, only 16% were censored, and yet similar treatment trajectories were observed. Fifth, regarding the high proportion of patients reducing the dose of their first TNFi over time, we cannot tell whether this is due to a recommendation by the physician and/or the patient’s own decision.

## Conclusions

A TNFi is the standard therapy for patients with active AS. It is therefore noteworthy that, while 54% of patients will have discontinued their first TNFi within 5 years, 63% are still treated with a biologic. Further, patients who do remain on a TNFi treatment appear to be able to reduce the dose over time, which may have vast implications for health economic evaluations of biologics in AS. These findings also highlight the need for thorough follow-ups to target patients with AS who are non-responsive to TNFi, and to determine predictors for such non-response, as well as alternative treatment options.

## Additional file

Additional file 1: **Table S1.** Calendar trends for number of patients with ankylosing spondylitis (AS) starting a first ever tumour necrosis factor alpha inhibitor year 2006-2015, with age, disease duration, BASDAI and ASDAS at treatment start, and *p* value for linear trend. As a comparison, the number of patients with rheumatoid arthritis (RA) starting a first bDMARD per year are also included. (DOCX 13 kb)

## Abbreviations

AS: Ankylosing spondylitis
ASDAS: Ankylosing spondylitis disease activity score
ATC: Anatomic therapeutic chemical
BASDAI: Bath Ankylosing Spondylitis Disease Activity Index
CI: Confidence interval
DDD: Defined daily dose
DMARD: Disease-modifying anti-rheumatic drug (biologic/conventional synthetic)
NSAID: Non-steroidal anti-inflammatory drug
RCT: Randomized controlled trial
SpA: Spondyloarthritis
SRQ: Swedish Rheumatology Quality Register
TNFi: Tumour necrosis factor inhibitors
