# Supplementary material for: Biological treatment of ankylosing spondylitis: a nationwide study of treatment trajectories on a patient level in clinical practice
Source: Arthritis Res Ther. 2019 May 28;21:128. doi: 10.1186/s13075-019-1908-9 (PMC6540538; doi:10.1186/s13075-019-1908-9)
Supplement: Supplementary file 1 — Table S1. Calendar trends for number of patients with ankylosing spondylitis (AS) starting a first ever tumour necrosis factor alpha inhibitor year 2006-2015, with age, disease duration, BASDAI and ASDAS at treatment start, and p value for linear trend. As a comparison, the number of patients with rheumatoid arthritis (RA) starting a first bDMARD per year are also included. (DOCX 13 kb) [file 13075_2019_1908_MOESM1_ESM.docx]

| Start year | 2006 | 2007 | 2008 | 2009 | 2010 | 2011 | 2012 | 2013 | 2014 | 2015 | P-value |
| --- | --- | --- | --- | --- | --- | --- | --- | --- | --- | --- | --- |
| Patients with AS N | 161 | 231 | 230 | 257 | 288 | 274 | 256 | 294 | 321 | 278 | 0.0036 |
| Patients with RA N | 971 | 964 | 1049 | 1177 | 1255 | 1262 | 1088 | 1220 | 1198 | 1138 | 0.0546 |
| Age mean (sd) | 43 (12.2) | 44 (12.3) | 43 (12.7) | 43 (13.0) | 44 (13.3) | 43 (13.2) | 42 (12.5) | 45 (13.8) | 44 (13.4) | 43 (14.3) | 0.9720 |
| Duration mean (sd) | 17 (12.2) | 16 (11.0) | 16 (11.9) | 15 (11.8) | 15 (12.2) | 16 (12.3) | 14 (12.1) | 17 (13.5) | 16 (12.8) | 15 (12.6) | 0.2489 |
| CRP mean (sd) | 24 (22) | 21 (26) | 15 (20) | 17 (25) | 19 (23) | 16 (19) | 15 (18) | 16 (21) | 17 (20) | 18 (26) | 0.0098 |
| BASDAI^1^ mean (sd) |  |  |  | 5.4 (1.93) | 5.5 (1.89) | 5.2 (2.11) | 5.3 (2.04) | 5.7 (2.12) | 5.2 (2.19) | 5.1 (2.04) | 0.2527 |
| ASDAS^1^ mean (sd) |  |  |  | 3.3 (1.03) | 3.5 (0.97) | 3.2 (1.03) | 3.2 (1.21) | 3.3 (0.99) | 3.3 (1.06) | 3.2 (1.03) | 0.4220 |
| 1) BASDAI and ASDAS are not included for 2006-2008 due to few registered values. | | | | | | | |  |  |  |  |

Additional file 1: Table S1 Calendar trends for number of patients with ankylosing spondylitis (AS) starting a first ever tumor necrosis factor alpha inhibitor year 2006-2015, with age, disease duration, BASDAI and ASDAS at treatment start, and p-value for linear trend. As a comparison number of patients with rheumatoid arthritis (RA) starting a first bDMARD per year are also included.
